# Epileptic spasms with terror during sleep in CDKL5 encephalopathy

**DOI:** 10.1093/sleepadvances/zpac010

**Published:** 2022-04-20

**Authors:** Gia Melikishvili, Artem Sharkov, Tamar Gachechiladze, Tatiana Tomenko, Alexandra Pivovarova, Iosif Volkov, Maria-Theresa Andrade, Abril Castellanos, Thierry Bienvenu, Olivier Dulac, Gabriel Roisman, Svetlana Gataullina

**Affiliations:** 1 Department of Pediatrics, MediClubGeorgia Medical Center, Tbilisi, Georgia; 2 Veltischev Research and Clinical Institute for Pediatrics of the Pirogov Russian National Research Medical University, Moscow, Russia; 3 Genomed ltd., Moscow, Russia; 4 Department of Brain Dysfunction and Epilepsy, Medical Center UMMC Health, Ekaterinbourg, Russia; 5 Cognitive-Behavioral Laboratory, Ural Federal University, Ekaterinbourg, Russia; 6 Department of Epilepsy, City Neurology Center “Sibneiromed” , Novosibirsk, Russia; 7 Instituto Hispalense de Pediatría, Sevilla, Spain; 8 Neurología Pediatría, Hospital Puerta de Hierro Norte, Zapopan, Jalisco, Mexico; 9 Laboratoire de Génétique et Biologie Moléculaires, Hôpital Cochin, AP-HP, Centre Université de Paris, Institut de Psychiatrie et de Neurosciences de Paris (IPNP), Inserm U1266 , Paris, France; 10 AdPueriVitam, Antony, France; 11 Service d’Explorations Fonctionnelles Multidisciplinaires, Centre de Médecine du Sommeil, Antoine Béclère Hospital, AP-HP, Clamart, France

**Keywords:** sleep terror, slow-wave sleep, quiet sleep, epileptic seizures, EEG, infant, child, epilepsy, clonazepam

## Abstract

**Study Objectives:**

To describe early diagnostic clues in Cyclin-Dependent Kinase-Like 5 (CDKL5) refractory encephalopathy, to improve treatment strategies.

**Methods:**

We retrospectively studied 35 patients (25 females, 10 males) with *CDKL5* gene mutations or deletion, focusing on their early seizure semiology, the electroencephalogram (EEG) pattern, the effect of treatment, and developmental outcome.

**Results:**

The first seizures were recognizable and consisted of tonic, then clonic, and spasms phases, occurring in sleep at a median age of 6 weeks. Clusters of spasms were observed in quiet sleep or slow-wave sleep (SWS), with screaming, staring, and arms’ extension that mimicked sleep terror in 28 of 35 patients (80%). Programmed awakening prevented these spasms in 9 of 16 patients and small doses of clonazepam given at night improved epilepsy in 14 of 23 patients.

**Conclusions:**

Peculiar seizures with spasms starting in SWS are an early diagnostic clue in infants with CDKL5 encephalopathy. Sleep video-EEG polygraphy is an easy tool to disclose these early seizures and epileptic spasms in infants during the first months of life while polysomnography is unlikely to give a contribution at that early age. While conventional antiepileptic treatment and corticosteroids are poorly, transiently, or not efficient, therapeutic strategy used for sleep terror could help, although the mechanism of spasms generation in SWS needs to be elucidated.

**Statement of Significance**

This series shows that, in contrast with the usual occurrence of epileptic spasms on awakening, spasms in Cyclin-Dependent Kinase-Like 5 (CDKL5) encephalopathy start from slow-wave sleep (or quiet sleep below three months of age), with a very peculiar sleep-related terror-like expression. Treatment strategies for sleep terror early in the course of epilepsy contribute to control seizures. Overexpression of GluN2B N-methyl-D-aspartate receptor subunit offers a potential target for the treatment strategy in CDKL5-related refractory epilepsy.

## Introduction

Cyclin-Dependent Kinase-Like 5 (CDKL5) encephalopathy is an X-linked disorder characterized by early-onset epilepsy with intractable seizures and severe psychomotor development delay, due to pathogenic variant (single nucleotide variant or large deletion) in *CDKL5* gene located on Xp22. It mainly affects female patients, although about 20% of males are on record, whose phenotype may be more severe. The deficient protein, serine/threonine kinase 9, controls several proteins associated with the neuronal skeleton [1]. It is likely that it regulates through this process the presence at the membrane of different proteins, including receptors and phosphorylation of splicing regulatory proteins, notably MeCP2. *CDKL5* being a *MeCP2*-repressing gene, respiratory and sleep disturbances characteristic of Rett syndrome are also seen in CDKL5 patients [2].

Moreover, the *CDKL5* gene may have a cytoplasmic function that regulates dendritic outgrowth and arborization [3,4]. Cdkl5 deficient mice show altered dendritic outgrowth and spine development. In this animal model, the formation of synapses remains at an immature stage [5], and the overexpression of GluN2B containing N-methyl-D-aspartate (NMDA) receptors causes neuronal hyperexcitability [6].

Epilepsy in CDKL5 encephalopathy being typically unresponsive to conventional therapy is associated with severe psychomotor development delay [7]. Early seizures occurring in the first year of life were qualified tonic-clonic, followed later by myoclonic seizures [8]. A closer subsequent observation led to the conclusion that these early seizures included an easily overlooked cluster of spasms, each one being preceded by a clonic jerk [9]. In addition, early spasms in CDKL5 infants occurred in slow-wave sleep (SWS), in contrast with those of other etiologies which usually occur on awakening or on falling asleep [10]. Terror-like events in sleep are seldom reported in CDKL5 encephalopathy and thus need to be distinguished from nocturnal seizures.

Here, as a means of proposing early therapeutic strategy based on possible common mechanism underlying seizures and sleep, our aim was to more accurately describe peculiar events that occur in sleep with sudden crying and behavior similar to that of sleep terror calling attention to an infant who is exhibiting a cluster of spasms.

## Methods

Our sample included 35 children (25 females, 10 males) with *CDKL5* single nucleotide variant or deletion. They came from Eastern Europe (Ukraine, Russia), Central Asia (Kazakhstan), Caucase (Georgia), Western Europe (Spain, France), and Mexico. Patients were identified on the basis of a characteristic clinical picture recorded on video and confirmed *CDKL5* mutation or deletion. Twenty-five of them were followed regularly by neuropediatricians from the countries listed above (authors G.M., A.S., T.G., T.T., I.V., M.T.A., A.C.) and referred to S.G. or O.D. for a second opinion on the early onset refractory epilepsy, whereas 10 others were diagnosed and subsequently followed by S.G. or O.D. (Table 1). Data were drawn in two steps: (1) Based on their files and additional parents’ interviews, we retrospectively analyzed clinical data, the semiology of both early and subsequent seizures, their link with sleep, home video, and ictal video-electroencephalogram (EEG) recordings data for all patients. (2) At the last visit performed for the sake of the study (Table 2), the interviews were conducted by the treating neuropediatrician (G.M., A.S., T.G., T.T., I.V., M.T.A., A.C., O.D., and S.G.) in charge of each patient, using a structured check-list established by S.G. Parents were asked the following standardized questions: age and daytime (sleep or awake) of the first seizure and its type, age when spasms were first noticed, time of the day of the spasms (sleep or awake), the time lag from falling asleep to the occurrence of sudden crying with a behavior similar to sleep terror with spasms, type of seizures at the last visit, developmental skills, sleep troubles, the effect of melatonin if used, that of each antiepileptic drug, especially the effect of combining zonisamide (ZNS) and vigabatrin (VGB), the effect of clonazepam (CZP) when given at night to control nocturnal seizures, and that of eventual programmed awakening. All data including EEG, video, and video-EEG were referred to the same person (S.G.)—neuropediatrician and neurophysiologist—for analysis and to determine the EEG pattern, namely presence or absence of hypsarrhythmia.

**Table 1.** Genetic features, epilepsy onset, and seizure semiology in the 35 CDKL5 encephalopathy patients

*N*, Sex	CDKL5 gene mutation or deletion	Age of first seizure	Type of first seizures		Spasms		Sleep terror
			Semiology	Nycthemeron	Age of first spasms	Nycthemeron	
1, Female	c.1153C>T p.(Gln385Ter)	8 Months	Sleep terrors like	Asleep	8 Months	Asleep	Yes until 5 years
2, Male	c.495dup p.(Ala166CysfsTer2)	4 Weeks	Spasms	Asleep	4 Weeks	Asleep	Yes
3, Female	c.464G>A p.(Gly155Asp)	3 Months	Spasms	Asleep	3 Months	Asleep	Yes
4, Male	c.283-25A>G	4 Months	Focal seizures	Awake and asleep	9 Months	Asleep	Yes
5, Male	c.226A>G p.(Lys76Glu)	6 Months	Spasms	Asleep	6 Months	Asleep	Yes
6, Female	c.655C>A p.(Gln219Lys)	3 Months	Clonic seizures with spasms	30 min after falling asleep	3 Months	Asleep	Yes at onset 3–5 months
7, Female	c.2673_2682del p.(Gln891HisfsTer23)	6 Weeks	Clonic	1 h after falling asleep	3 Months	Asleep	Yes
8, Female	c.1952_1955dup p.(Leu653ThrfsTer31)	4 Weeks	Sleep terrors like	35 min after falling asleep	6 Months	Asleep	Yes
9, Female	c.2203_2233dup p.(Ser745)*	2 Months	Clonic or myoclonic in sleep	Asleep	10 Months	Asleep	Yes
10, Female	c.404-1G>T	4 Months	Tonic, then clonic, then spasms	Asleep	7 Months	Asleep	Yes until 2 years
11, Male	c.148-1G>A	2.5 Months	Tonic, then clonic, then spasms	15–20 min after falling asleep	7 Months	15–20 min after falling asleep	Yes
12, Female	c.521C>A p.(Thr174Asn)	3 Weeks	Tonic, then clonic, then spasms	10 min after falling asleep	3 Months	30 min after falling asleep	No
13, Female	c.578A>T p.(Asp193Val)	4 Weeks	Tonic, then partial clonic, then bilateral asynchronous myoclonus	Asleep	10 Weeks	Asleep	Yes
14, Female	c.1734_1738dup p.(Ser580Thrfs*38)	6 Weeks	Tonic, then focal clonic then bilateral asynchronous myoclonus with spasms	5 min after falling asleep	11 Weeks	2–10 min after falling asleep	No
15, Female	c.1008_1030del p.(Ser337GlyfsTer13)	1 Month	Spasms	Asleep	4 Months	8–15 min after falling asleep	No
16, Female	c.119C>T p.(Ala40Val)	7 Weeks	Tonic, then clonic	Awake	20 Weeks	Awake and asleep 2–20 min after falling asleep	Yes
17, Male	c.212A>G p.(Asn71Ser)	7 Weeks	Partial clonic	Awake	6 Months	Asleep	Yes
18, Female	c.1151dup p.(Tyr384Ter)	2 Weeks	Clonic	Asleep	2 Weeks	10–15 min after falling asleep, then 1 h after falling asleep at 5 years	Yes from 6 months to 3 years
19, Female	c.2635_2636del p.(Leu879GlufsTer49)	4 Months	Partial clonic	15 min after falling asleep	4 Months	Awake and asleep	Yes
20, Female	exon 6 deletion NC_000023.10:g.18592659_18594590del	3 Months	Eyelids myoclonus	15 min–3 hours after falling asleep, then every 2 h	4 Months	Asleep	Yes
21, Female	c.2432del p.(Ala811ValfsTer26)	1 Month	Eyelids myoclonus	Asleep	2.5 Months	Asleep	Yes
22, Female	c.1589dup p.(Thr531AsnfsTer7)	1 Month	Spasms	Falling asleep	1 Month	Sleep	No
23, Female	deletion 50 bp Xp22.11	2 Weeks	Spasms	Asleep	2 Months	On falling asleep	No
24, Female	c.554 + 1G>A	1 Month	Tonic	Falling asleep	5 Months	1 h after falling asleep	Yes/between 6 and 13 months
25, Female	c.527G>A p.(Trp176Ter)	2 Weeks	Tonic followed by spasms	Falling asleep	2 Months	30 min after falling asleep	Yes
26, Female	c.506_507del p.(Thr169ArgfsTer36)	1.5 Months	Clonic followed by spasms	Asleep	2 Months	Falling asleep	Yes
27, Female	NC_000023.10:g.18476781_18490917del	3 Months	Spasms	Asleep	4 Months	Asleep	Yes
28, Female	c.454T>C p.(Cys152Arg)	1.5 Month	Tonic	Asleep	3 Months	15–20 min after falling asleep	Yes
29, Female	c.1018A>T, p.(Arg340Ter)	3 Months	Spasms	Asleep	3 Months	Asleep	Yes
30, Male	c.349dup p.(Tyr117LeufsTer12)	1 Month	Spasms	Asleep	1 Month	Awake and asleep	No
31, Female	с.1486A>T, p.(Lys496Ter)	1 Month	Tonic with left side head version	Asleep	7 Months	Asleep	Yes
32, Male	c.400=/C>T, p.(Arg134Ter)	6 Months	Spasms	Asleep	6 Months	Awake and asleep	No
33, Male	c.744 + 1=/G>A p.(?)	1 Month	Spasms	Both	1 Month	Asleep	Yes
34, Female	с.1178_1179insTGGGGCAGCTA p.(Ser394GlyfsTer103)	2.5 Months	Tonic	Both	5 Months	Awake and asleep	Yes
35, Male	c.2413C>T, p.(Gln805Ter)	2 Months	Spasms	Asleep	4 Months	Awake and asleep	Yes

**Table 2.** EEG pattern, rational treatment effect, developmental outcome, and seizure state at the last visit in the 35 CDKL5 encephalopathy patients

No.	Hypsarrhythmia	Effect of CZP	Effect of VGB +ZNS	Prevention with awakening	End of follow-up, age	Developmental outcome	Persisting seizures at the end of the follow-up
1	No	Not given	No effect	Yes	6 Years	Moderate delay: walks, speaks	Spasms 4–5/day
2	Yes	Reduction by over 75%	Remission 1 year	NA	8 Years	Severe delay: does not sit nor walk	Daily myoclonic seizures followed by hypomotor phase then myoclonic spasms
3	Yes	No effect	Remission 1 year	NA	14 Years	Sev	Bilateral myoclonus combined with spasms
4	Yes	Remission 12 months	Worsening	NA	9 Years	Severe delay: severe hypotonia, does not sit nor walk	Tonic or myoclonic spasms 4/week
5	Yes	No effect	Not given	NA	3 Years	Severe delay: severe hypotonia, does not hold his head nor sit	Rare spasms in sleep
6	No	Remission 8 months	Remission 3 months	Yes	2 Years 2 Months	Moderate delay: stands, walks with help	Daily spasms in sleep
7	Yes	No effect	Remission 5 months	NA	5 Years	Severe delay: hypotonia, does not sit nor walk	Up to 10 spasms per day, anytime
8	No	No effect	No effect	Yes	2.5 Years	Moderate delay: can sit, does not walk	Clusters of spasms in sleep
9	Yes	Not given	Not given	NA	5 Years	Severe delay: can sit, does not walk	Bilateral myoclonic seizures combined with spasms, 3–4/months
10	Yes	Not given	Partial	NA	5 Years	Severe delay: does not sit nor walk	Daily spasms, bilateral tonic with hyperkinetic automatisms
11	Yes	Not given	Not given	Yes	4 Years	Severe delay: does not sit nor walk	Spasms in sleep each night or nap, that can be prevent by programmed awakening
12	Yes	Reduction by over 75%, then 4 months’ remission	Remission 3 months	No effect	2 Years	Severe delay: hypotonia, does not sit nor use her hands	Daily spasms
13	Yes	Remission 4 months	Remission 5 months	Yes	5.5 Years	Severe delay: hypotonia, does not stand nor walk	Spasms in sleep, weekly
14	Yes	No effect	Remission 5 years	NA	6 Years	Moderate delay: walks with help, does not speak	Seizure-free since 1 year of age with VGB + ZNS
15	Yes	No effect	ZNS alone over 50% reduction of seizures	NA	10 Years	Severe delay: does not stand nor sit	Rare spasms and seizures with tonic posturing of right limbs followed by clonic movements of right face, awake
16	NA	No effect	Remission 3 years	NA	14 Years	Severe delay: no acquisition	Spasms once a week
17	No	No effect	Remission 6 months	NA	13 Years	Moderate delay: walks, does not speak	Seizure-free since the age of 10 years with VPA + PHT
18	Yes	Reduction by over 75%	Not given	NA	6 Years	Moderate delay: walks, does not speak	Spasms in sleep, once a week
19	No	Reduction by over 75%	No effect	NA	2.5 Years	Moderate delay: can sit and stand	Terror-like spasms in sleep once a month
20	No	Remission 5 months, then partial effect	Not given	NA	4 Years	Moderate delay: sits, does not walk nor speak	Tonic phase, then myoclonic spasms
21	No	Reduction by over 75%	Not given	Yes	2 Years	Moderate delay: sits, does not walk	Spasms awake, 2 to 3 a month
22	No	No effect	Not given	No effect	12 Months	Moderate delay: does not sit	Sleep spasms each nap, once a day
23	No	Remission (CLB)	Not given	No effect	14 Years	Mild delay: walks, talks	Seizure-free since the age of 1 year (no more treatment)
24	No	Remission more than 12 months	Remission 8 months	Yes	9 Years	Moderate delay: stands, does not walk	Seizure-free since the age of 8 years on CZP
25	No	Reduction by over 90% of sleep seizures	VGB alone over 90% reduction	Yes	15 Months	Severe delay: does not sit, smiles	Rare terror-like spasms in sleep, 2 per week, on CZP at night
26	NA	Not given	ZNS alone, remission 6 months	Yes	10 Years	Moderate delay: walks but does not speak	Rare myoclonic seizures or myoclonic spasms preceded by tonic phase then hypomotor phase in sleep on VNS, ZNS, LVT
27	Yes	Not given	Not given	NA	5 Years	Severe delay: sits, does not stand	Daily spasms, mainly awake
28	No	Not given	Not given	No effect	12 Months	Moderate delay: does not sit	Daily tonic followed by spasms in sleep
29	Yes	Not given	Worsening	No effect	5 Years	Severe delay: sits but does not walk	Spasms and tonic seizures in sleep
30	Yes	Not given	Not given	No effect	14 Years	Severe delay: does not walk, does not speak	Daily bilateral tonic and myoclonic in sleep
31	Yes	Remission 6 months	Not given	NA	4 Years	Severe delay: does not sit, does not walk	Daily bilateral tonic/atonic/clonic
32	Yes	Not given	Not given	NA	3 Years	Moderate delay: sits, does not walk	Bilateral tonic with hyperkinetic automatisms, every 2 days
33	No	Not given	Not given	NA	5 Years	Moderate delay: does not walk	Bilateral tonic-clonic, every two days
34	Yes	Not given	Not given	NA	5 Years	Severe delay: does not sit nor walk	Spasms, myoclonic seizures, every two days
35	Yes	Reduction by over 50%	No effect	No	12 Months	Severe delay: no eye contact, does not sit	Tonic seizures and spasms

NA, Not available; CLB, clobazam; CZP, clonazepam; LVT, levitiracetam; PHT, phenytoin; VGB, vigabatrin; VNS, vagal nerve stimulator; VPA, valproic acid; ZNS, zonisamide.

Five patients with incomplete clinical data were excluded: namely those with no available video or EEG-video recordings, and no access to regular follow-up. The follow-up period lasted from 12 months to 14 years (mean = 6 years).

A list of single nucleotide variants and deletions is provided in Table 1. Eleven mutations were reported earlier (http://mecp2.chw.edu.au/cdkl5/cdkl5_home.php). Three boys had mosaicism, one of whom had a typical clinical picture while another carried a variant of *CDKL5* considered of uncertain significance, inherited from his asymptomatic mother with no X-inactivation bias (Table 1: patient No. 4).

A nonparametric Mann–Whitney test was used to compare the age of epilepsy onset of infants presenting with and without terror-associated spasms.

## Results

### Seizures

#### *First seizure*s.

The parents were the ones who noticed the first seizures. They occurred at the median age of 6 weeks (range 2 weeks to 6 months, standard error of the mean [SEM] = ±5 weeks) and during sleep for the majority (31/35, 89%). A peculiar sequence described previously in CDKL5 patients affected 30/35 patients: a tonic phase was followed by a brief hypomotor phase, and then a clonic phase with a cluster of very mild spasms that, unless recorded on video-EEG polygraphy, were often overlooked (Supplementary Video 1). The clonic component was pronounced from the onset and included eyelid jerks. Five other patients commenced with spasms at the later median age of 2 months (range 4 weeks to 8 months, SEM = ±2 months).

#### *Spasms*.

**Figure 1.**
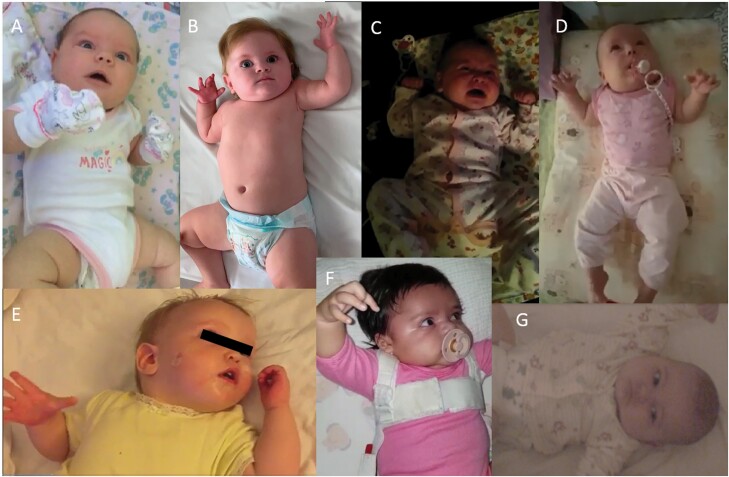
Mosaic of screenshots from several videos showing seizures in sleep mimicking night terrors. (A) Patient 12 at the age of 1 month. (B) Patient 8 at the age of 4 months. (C) Patient 29 at the age of 3 months. (D) Patient 25 at the age of 2 months. (E) Patient 1 at the age of 8 months. (F) Patient 22 at the age of 1 month. (G) Patient 26 at the age of 2 months. Notice common facial expression with staring, crying, and arms extension in all patients.

**Figure 2.**
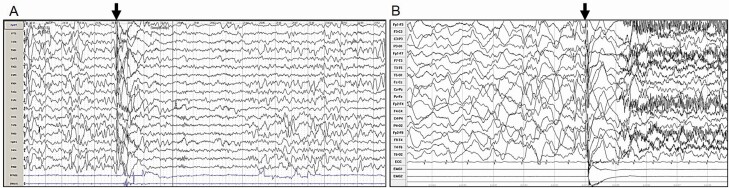
Ictal EEG recordings. (A) Ictal EEG showing a spasm occurring in SWS in patient no. 15 at 1 year and 10 months of age. Sweep 15 mm/s, sensitivity 20 µV/mm. Bi-polar longitudinal montage. (**B**) Ictal EEG showing a sleep spasm in patient no. 18, at 5 years of age, starting from slow-wave sleep. Sweep 30 mm/s, sensitivity 10 µV/mm. Bi-polar longitudinal montage. Notice a recruitment pattern characteristic of epileptic spasm indicated by arrow in both patients, associated with deltoids contraction visible on the surface electromyogram (EMG).

Clusters of spasms became more evident with age and were first noticed by the parents at a mean age of 4 months (range from 1 to 10 months, SEM = ±1.8 months). In all patients, they occurred during nap time or night sleep, starting between 5 min and an hour after falling asleep, in other words well before spontaneous awakening. For 28 of 35 patients (80%), they started with sudden awakening, commencing with subtle and easily overlooked extension of the arms followed by staring, and then crying and yelling, clearly mimicking sleep terror (Figure 1; Supplementary Video 2). In three patients, EEG recording of this sequence showed that it was entirely epileptic. Nap time video-EEG polygraphy revealed epileptic spasms starting from SWS (Figure 2). We were surprised to learn that epilepsy started slightly later in infants presenting with terror-associated spasms (median age of onset of fist seizures = 2 months, *n* = 28) than in those without terror-like epileptic events (median age = 1 month, *n* = 7), although not at a significant level (*p* = .08).

#### *Subsequent seizures*.

Spasms in clusters persisted until the end of the follow-up (14 years for the oldest patient). From the middle of the first decade, however, seizures were less linked to sleep in general although while they persisted in nocturnal sleep, they mainly occurred when the child was awake. They consisted of a variable combination of myoclonic and tonic phases accompanied by a cluster of spasms. The myoclonic component persisted throughout the evolution of the seizures combined with spasms, even in the late stage of the disease. It is particularly noteworthy that the care givers themselves noticed that, in contrast with the generalized tonic-clonic seizures, the child still retained eye contact during these complex seizures, thus ruling out a loss of consciousness.

### Electroencephalography

#### *Ictal EEG*.

Epileptic spasms were recorded in five patients. In all five, spasms started from QS (below 3 months of age) or SWS (above 3 months of age). The epileptic spasms were accompanied by a characteristic recruitment pattern (Figure 2). The initial tonic component with sudden awakening and extended arms during terror-associated spasms was not, however, accompanied by any ictal pattern but a myographic artifact, suggesting a possible subcortical origin. By contrast, this tonic phase was followed by a pattern of typical spasms with recruitment pattern.

#### Interictal EEG.

Interictal EEG was available for all patients. For over half (20/35, 57%), interictal EEG showed hypsarrhythmia on repeated tracings and during the first 3 years of the disease with steroids being able to stop it in barely half of them (Table 2). For the others, few to no multifocal spikes were observed. Normal SWS pattern with bi-central spindles of 13–14 Hz, preceded the onset of spasms occurring during sleep.

### Effect of medication

Twenty patients were given a combination of ZNS (6 mg/kg/d) and VGB (100 mg/kg/d). Eleven (55%) had partial effect or transient seizure arrest lasting 3 to 12 months, while three others became seizure-free for up to 5 years, one of whom was still seizure-free at the end of the follow-up. Two, however, experienced a seizure increase while four had no effect at all. Conventional antiepileptic drugs like valproate, topiramate (TPM), perampanel, and rufinamide, resulted in a partial improvement, with transitory seizure freedom rarely occurring. During the first 2 years of the disease, hormonal treatment by hydrocortisone (HC) or ACTH was given to 11 patients (9 of them having hypsarrhythmia), the transient seizure-free period lasting from three weeks to one year. In contrast, steroids aggravated seizures in three patients who had daily spasms but no hypsarrhythmia.

A worsening of antiepileptic medication was occasionally observed, namely in conjunction with the use of carbamazepine, oxcarbazepine, TPM, levetiracetam, sultiam, and ZNS (one patient each).

Paradoxically, sodium channel inhibitors, phenytoin (PHT), and carbamazepine, relieved seizures in two patients who had no hypsarrhythmia, one of whom were found to be seizure-free at the last visit. Given its known efficiency in sleep terrors, small doses of CZP (drops, 0.1 mg/kg) were given at night to 23 patients. For 14 (61%) patients, seizures in sleep were significantly reduced (over 75% decrease) or entirely stopped for four to 12 months. In addition, for three patients, their parents noticed an improvement of sleep on CZP. Programmed awakening ensured by parents prevented or attenuated nocturnal spasms in over half of the patients (9/16). It is interesting that in most instances, the parents themselves noticed that programmed awakening could help. This observation was mentioned in several files and was part of the last visit interview checklist.

At the end of the follow-up period, four patients were seizure-free, two were on CZP, one on combination of ZNS and VGB, and one on PHT.

### Sleep disturbances

*Sleep disturbances* were reported for at least half (13) of the patients, taking the form of frequent awakenings at night, inversion of the circadian rhythm, and insomnia. Melatonin given to 10 patients, had no effect on sleep, save for one who received the slow release form (Circadin).

### Developmental outcome

At the end of the follow-up period that ranged from 12 months to 14 years, 6 of the 13 patients aged from 12 months to 5 years could sit, while 6 of the 13 aged from 5 to 14 years could walk. Only two patients aged 6 and 14 years could say sentences. Twenty-four patients were severely developmentally delayed with poor eye contact and major hypotonia. All those who had long-lasting hypsarrhythmia had poor development, being unable to sit or stand. Five of the six patients (83%) who could walk had never had hypsarrhythmia on repeated EEGs.

Boys having complete *CDKL5* mutation had no developmental skills and remained severely hypotonic and hypotrophic, while the three boys (patients No. 17, 32, 33, Tables 1 and 2) with a mosaic mutation had better development than girls. The boy with an inherited variant of unknown significance inherited from his mother had severe developmental delay with no acquisition and major hypotonia (patient No. 4, Tables 1 and 2).

## Discussion

This series shows that, based on the semiology of terror-associated spasms, CDKL5 encephalopathy could be recognized very early in the course of the disease. Spasms are the most frequent seizure type in this disorder often without hypsarrhythmia [11]. In a study of 24 patients with infantile spasms of various etiologies, Kellaway *et al*. [10] showed that the clusters predominantly occurred soon after awakening. In contrast, seizures in CDKL5 encephalopathy typically start in QS or SWS, accompanied by stark terror and screaming until brisk movements upper limbs allow to recognize the spasms. Here we report as a specific diagnostic clue not reported to date, namely that these particular spasms start in QS or SWS, several of which were considered as very unusual sleep terrors, because they occur during the first years of life.

### Spasms in sleep as a diagnostic clue

Epileptic spasms in CDKL5 encephalopathy are often difficult to recognize in the beginning, particularly as regards the sequence of early seizures. Their characteristics suggest CDKL5 encephalopathy, which enables a selective targeting of this gene, thereby saving precious weeks or months of searching. Interestingly, the age of onset was found to be later for patients with terror-associated spasms in contrast with those without, indicating that a degree of sleep maturation may be a precondition for generating such events.

Sleep troubles are known to affect more than half of the patients with *CDKL5* mutations. Despite awakening with screaming spells, their epileptic nature was not suspected. Furthermore, in patients with CDKL5 encephalopathy, seizures mainly in sleep have been reported in a few cases whereas the link with SWS has not been mentioned nor have spasms linked to sleep terror-like events [12,13].

Spasms occurring in SWS were documented in the case of five patients by the means of ictal EEG, and for the others, parents clearly stated that the seizures started during sleep, and that they mimicked sleep terror. Indeed, with the exception of three who were awake, the first seizures started only when patients were asleep. Parents were able to take notice of the precise delay between the time their child fell asleep and the moment when spasms occurred, thus making it possible for them to prevent them by awakening the child. Occurrence of spasms within the first hour after falling asleep suggests that they started from SWS. Clinically, child behavior indicates QS before three months with SWS coming later (stage 2 or 3), and in five cases, this was confirmed by ictal EEG. At this young age, during transition stages of early sleep, it is difficult to distinguish whether the infant is in a stage 2 or stage 3 of SWS although sleep spindles were obvious preceding spasms onset. Although polysomnography is therefore believed unlikely to be helpful in treating infants in the early stages of the disease, it could nevertheless bring further valuable information regarding sleep organization later in life.

Early-onset spasms starting from SWS have never been identified as a specific diagnostic clue in infants with CDKL5 encephalopathy. It has been shown in a *Cdkl5* mice model, however, that clusters of spasms can similarly occur during SWS, accompanied by a characteristic EEG background activity and elevated delta power [14].

According to the American Academy of Sleep Medicine’s *International Classification of Sleep Disorders,* sleep terror in healthy children is characterized by sudden awakening from SWS with “a cry or piercing scream, behavioral manifestations of intense fear and confusion accompanied by autonomic nervous system signs” including sweating and increased heart rate. Sleep terror in healthy children begins at a far older age than do seizures in CDKL5 encephalopathy, with a peak between ages 18 months and 4 years (for a review, see Leung *et al*. [15]). Confusion between the two seems unlikely.

### Genetics

Most patients had de novo mutations. However, for one boy (patient 4), a rare CDKL5 intronic variant c.283-25A>G was inherited from his asymptomatic mother. Variants transmitted by asymptomatic mothers are rare and often the pathogenicity of the variant is not demonstrated [16]. Nevertheless, this very rare variant was only reported in seven alleles out of 111 682 in the gnomAD database (https://gnomad.broadinstitute.org/) and only in heterozygous individuals. It showed a CADD phred score of 13.33 (https://cadd.gs.washington.edu/) that might influence the splicing process by creating a cryptic splice site within intron 5 of the *CDKL5* gene. mRNA analysis to show that this variant disrupts normal splicing of intron 5 could not be performed. Therefore, in the present case, the clinical symptomatology we report here is particularly important to reach the proper diagnosis and avoid excluding this gene.

### EEG findings

In contrast with the ictal features being a clue for the diagnosis, the interictal EEG may be misleading: following the first seizures, it may show no abnormalities or only slow background activity with few or no spikes [17]. While it is common practice to repeat the EEG if the first tracing has failed to show any spikes, the suspicion of sleep terror is not by itself, sufficient justification for doing so. In our study as in others [9], evidence of hypsarrhythmia was recorded in barely half the cases, when spasms had become obvious in the middle of the first year of life. In these cases, the prognosis was poor.

### Possible pathways underlying the spasms in CDKL5 encephalopathy

An early onset with an ictal sequence starting in SWS with a tonic event and spasms suggests a possible mechanism underlying the epilepsy in CDKL5 encephalopathy. SWS is initiated by both glutamate and gamma-amino-butyric acid (GABA) transmissions and is under the control of the ventro-lateral preoptic nucleus (VLPO). It is during SWS that these two neurotransmitters reach their highest level in the whole sleep-wake cycle [18]. Glutamatergic excitatory neurotransmission is upregulated in CDKL5 encephalopathy, due to both alpha-amino-3-hydroxy-5-methyl-4-isoxazolepropionic acid (AMPA) receptor dysregulation and GluN2B NMDA-receptor (NMDA-R) subunit overexpression [6,19]. GluN2B NMDA-R has age- and region-specific distribution, the main expression being in the spinal cord, the brainstem and around the thalamus [20]. It is therefore in sleep that an overexpression of GluN2B NMDA-R is the most likely to have pathogenic impact, particularly in SWS, in which glutamate is the greatest contributor.

The pontine intermediary reticular formation that activates the axial musculature, is linked to both the spinal cord and the hypothalamus, including the supra-optic nucleus. It could, therefore, generate a tonic component for which there is no EEG counterpart, and that triggers the seizure in SWS, particularly in the early stage of the epilepsy.

Based on a positron emission tomography analysis, Chugani *et al*. [21] suspected that the brainstem was responsible for generating epileptic spasms. This seems indeed to be the case in CDKL5 encephalopathy. Respiratory troubles reported in this condition also point to brainstem dysfunction. Sleep apneas are more common in *Cdkl5*-KO than in wild-type mice [22]. From this, we conclude that, in addition to the cortex, the insula and the lenticulum that all contribute to generate epileptic spasms [23, 24], the brainstem thus seems to be another important structure possibly implicated in this condition.

Although epilepsy typically commences in the first weeks of life, 90% being before three months, it does not begin in the neonatal period [25], which suggests that some maturation process is a necessary precondition. Switching from a neonatal GluN2B NMDA subunit to an adult GluN2A and the inversion of GABA current are the most likely intervening variables since they take place in the first weeks of life [20, 26]. On the one hand, the combination of an excess of NMDA and excitatory GABA transmissions seems to contribute to neonatal myoclonic encephalopathy and West syndrome [27,28]. On the other hand, CDKL5 epilepsy starts following the neonatal period with median age of 1.5 month, once GABA transmission particularly involved in sleep has become inhibitory.

The combination of spasms and myoclonus is another striking ictal feature in CDKL5 encephalopathy that persists throughout the course of the disease. The combination of spasms and myoclonus becomes more and more evident as time goes by [11]. Asynchronous myoclonus, affecting limbs and eyelids before each spasm, could be a triggering factor. However, the genesis of myoclonus remains to be identified.

**Figure 3.**
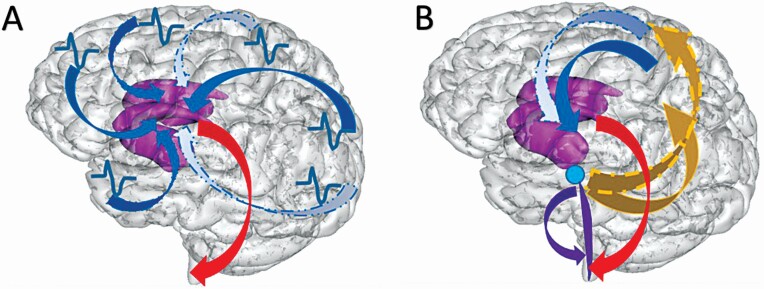
Schematic representation of pathways possibly involved in the generation of cryptogenic spasms versus spasms in CDKL5 encephalopathy. (A) *Cryptogenic spasms:* diffuse hyperexcitability of the cortex activates motor programs in the lenticular nucleus (efferent pathways from the cortex), generating the spasms (efferent pathway from the lenticulum). (B) *CDKL5 encephalopathy*: during a SWS induced by ventro-lateral preoptic nucleus (VLPO), excitatory neurotransmission mediated by upregulated GluN2B NMDA receptors in the brainstem will activate reticular-spinal pathway inducing the tonic event (efferent pathways in the brainstem), then the cortical motor strip (afferent pathways to the motor strip) causing myoclonic jerk, then the lenticular nucleus (efferent pathways from the motor strip), generating the spasms (efferent pathway from the lenticulum).

In the premature infant, spontaneous myoclonus generated by spinal motor neurons triggers, in central areas, through sensory pathways, delta activity with fast oscillations called delta brush [29, 30]. Visual and auditory stimuli trigger the same pattern in occipital and temporal areas, respectively [31, 32], contributing to the cortical map formation and function. Delta brush is driven mainly by glutamatergic NMDA receptors’-mediated transmission while GABA inhibition determines their compartmentalization [33, 34]. However, since the expression of GluN2B NMDA-R starts from birth, its overexpression could disturb cortical maturation and maintain this triggering process in the immature state beyond the neonatal period, contributing to the persistence of florid myoclonic activity for many years. Therefore, when the VLPO generating SWS activates reticular formation, the excessive excitatory neurotransmission mediated by overexpressed GluN2B containing NMDA-R would activate both the reticulo-spinal pathway (triggering a tonic event) and the cortical motor strip. This would provoke a jerk which could in turn trigger the lenticular nucleus and produce the spasm (Figure 3). Being expressed mainly in the brainstem, overexpression of GluN2B could contribute to alter sleep organization and maturation.

A common mechanism in CDKL5 encephalopathy and night terror can be suspected. Indeed, 5-hydroxytryptophane, a serotonin precursor, was shown to suppress night terrors in children [35] while citalopram, a serotonin reuptake inhibitor suppresses physiological twitches driving spindle burst (equivalent of delta brush) [36] as myoclonus may drive a spasm. This could add some rational to using serotoninergic therapy, particularly since fluoxetine was shown to selectively inhibit the GluN2B NMDA-R subunit [37].

### Treatment strategies

Recalling that any treatment strategy must consider the sequence of epilepsy, it is emphasized that there is no antiepileptic medication to date that has a long-lasting effect in CDKL5 encephalopathy, be it VGB, TPM, and ZNS monotherapies [25, 38]. ZNS combined with VGB can reduce seizures [9], especially when given early. Since, however, the effects of conventional antiepileptic medication are, at best, no more than transient, any trial with a new compound should be prospectively controlled although be it said that, given the scarcity of this condition, they rarely are [39].

The effect of hormonal therapy is similarly transient, occurring when hypsarrhythmia is present. Neither the ketogenic diet, nor vagal nerve stimulation, nor even callosotomy have more than an anecdotal effect [25]. To note, however, is the case of two patients without hypsarrhythmia, who were given sodium channel inhibitors, namely carbamazepine and PHT, which greatly improved their condition whereas, when they were given to those with hypsarrhythmia, the condition worsened. Studies of animal data support the view that increasing pharmaco-resistance may be a consequence of synaptic damage appearing in the course of the disease, possibly as a consequence of GluN2B-mediated excitotoxicity, in analogous to an inborn error of metabolism [4, 40]. It is hoped that this finding will encourage the development of specific compounds that, by targeting the recently disentangled mechanism, will contribute to the reduction of NMDA transmission.

The alternative to conventional treatment consists of focusing on the events that simulate sleep terror. Benzodiazepines have been found to be effective in reducing the frequency of sleep-related seizures in CDKL5 encephalopathy, just as they do for sleep terror [41]. They have been shown to control sleep spasms, allowing long-lasting remission either because they reduce the depth of SWS or because of an additional antiepileptic effect. An alternative practiced by some parents is to reduce the depth of SWS and prevent the seizures by waking the child up a few minutes before the usual time of seizure occurrence. Although such a solution may seem challenging for both the caretaker and the child for the inescapable insomnia and sleep disturbance it causes, many parents conclude that the risk/benefit ratio is favorable.

As for developmental skills, they were found to be very slow in girls and almost absent in two boys, a finding consistent with more severe phenotypes having been found in boys than in girls [42] unless the disease is caused by mosaic mutation.

### Limitations

This study is based on the data obtained from a retrospective chart review, and it is clear that a much larger sample is required along with an analysis of prospective video-EEG recordings to increase the reliability of the findings.

## Conclusion

An early diagnosis of CDKL5 encephalopathy is clinically feasible based on the discrete characteristics of sleep-related seizures. Sleep terror-like events in infancy linked to spasms could be a diagnostic clue to new pathways for specific treatment, including therapeutic strategy commonly applied to sleep terrors.

## Supplementary material

Supplementary material is available at *SLEEP Advances* online.

Supplementary Video 1. Video EEG-polygraphy showing a typical early seizure in patient 14, at 2 months and 24 days of age, starting from quiet sleep with initial myoclonic then tonic phase followed by right clonic phase and myoclonic spasms. Sweep 30 mm/s, sensitivity 10 µV/mm, bipolar montage.

Supplementary Video 2. Typical seizure mimicking sleep terror in patient 8, at the age of 4 months, 30 min after falling asleep (home video).

zpac010_suppl_Supplementary_Video_S1

zpac010_suppl_Supplementary_Video_S2
